# Impact of acidic pH and sealer thickness on the physical behavior of endodontic sealers: a micro-CT study

**DOI:** 10.1590/1807-3107bor-2026.vol40.028

**Published:** 2026-06-01

**Authors:** Karina Ines Medina Carita TAVARES, Airton Oliveira SANTOS-JUNIOR, Jáder Camilo PINTO, Fernanda Ferrari Esteves TORRES, Juliane Maria GUERREIRO-TANOMARU, Mário TANOMARU-FILHO

**Affiliations:** (a) Universidade Estadual Paulista – Unesp, School of Dentistry, Department of Restorative Dentistry, Araraquara, SP, Brazil.; (b) Centro Universitário Presidente Antônio Carlos - Unipac, Departament of Dentistry, Barbacena, MG, Brazil.

**Keywords:** Dental Materials, Physical Phenomena, Root Canal Obturation, X-Ray Microtomography

## Abstract

The aim of this study was to evaluate the effect of immersion in an acidic (butyric acid – BA; pH 4.1) or neutral (phosphate-buffered saline - PBS; pH 7.0) environment on the physical behavior of Bio-C Sealer (BCS) or AH Plus Jet (AHPJ) after filling oval root canals with different thicknesses of sealer. Thirty-two oval root canals were prepared and filled with BCS or AHPJ (n = 16), using the single-cone technique. Greater, middle, and smaller thicknesses of sealer were established in the cervical, middle, and apical thirds of the root canals, respectively. Then, the samples were immersed in BA or PBS (n = 8) for time intervals of 7 and 30 days. Micro-CT scans were performed after the materials set and after the experimental time intervals ended. Volumetric change and the percentage of voids and gaps at the material/dentin interface were evaluated. Kruskal-Wallis and Dunn, Friedman, Wilcoxon paired, and ANOVA/Tukey tests were performed (α = 0.05). Significant volumetric change and percentage of voids and gaps at the interface of BCS and AHPJ were observed in BA after 30 days (p < 0.05). Both materials showed a higher percentage of voids and volumetric loss in the greater sealer thickness (p < 0.05). Baseline showed a lower percentage of voids and gaps at the interface when compared with the experimental periods (p < 0.05). An acidic environment harms the properties of endodontic sealers after 30 days. The greater sealer thickness negatively affected the volumetric change and presence of voids in oval root canals.

## Introduction

Three-dimensional filling of root canals is essential, especially in root canals with anatomical complexities.^1,2^ Among the filling techniques, the single-cone type is considered easy to perform,^3^ but a greater amount of sealer is used for adequate filling of the root canals.^3,4^ Therefore, the properties of endodontic sealers are even more important since their volumetric behavior, filling capacity, and interaction with dentin can impact the success of endodontic treatment.^1,2,4-7^

Bio-C Sealer (BCS; Angelus, Londrina, Brazil) is a pre-mixed, ready-to-use sealer with properties that comply with the ISO 6876:2012 requirements,^5^ including setting time, radiopacity, flow, alkaline pH, and low volumetric change by micro-computed tomographic analysis (micro-CT).^8^ Furthermore, BCS showed a low percentage of voids, similar to those in AH Plus (AHP; Dentsply DeTrey GmbH, Konstanz, Germany),^2^ in addition to a low presence of marginal gaps when compared with AH Plus Jet (AHPJ; Dentsply).^7^ However, BCS does not comply with the solubility values stipulated by ISO 6876.^5,8,9^ AHPJ is an epoxy resin-based sealer available in paste-paste form in an automix syringe. This material is usually used as a comparison standard for investigating new sealers^2,8,10^ due to its excellent physicochemical properties,^8,10^ including low solubility.^9,10^

The physicochemical behavior of endodontic sealers can be affected in an acidic environment.^11-15^ Acidic pH derived from the by-products of inflammation in the presence of apical periodontitis has been shown to change the chemical composition and damage the hydration reaction of ProRoot MTA cement (Dentsply Maillefer, Ballaigues, Switzerland), Biodentine (Septodont, Saint-Maur-des-Fossés, France), CEM Cement (Bionique, Tehran, Iran) and Retro MTA (BioMTA, Seoul, Republic of Korea).^12^ Furthermore, an acidic pH increases the volumetric loss of Biodentine,^11^ Bio-C Repair (Angelus)^13^ and EndoSequence BC Sealer (Brasseler, Savannah, USA),^1^ and promoted lower bond strength for EndoSequence Root Repair Material (Brasseler).^14^ In contrast, the use of simulated body fluid (phosphate-buffered saline - PBS) has been shown to decrease the solubility and volumetric change of calcium silicate-based materials.^1,5,9,13^ Furthermore, its similarity to a clinical application can help with understanding the physical behavior of root canal sealers.^5,9^

Sealer thickness is another factor that influences the quality of the root canal filling.^4,16^ A smaller thickness of sealer between the gutta-percha and root canal wall is considered ideal.^3,17^ Generally, the cervical third requires a larger amount of sealer during filling,^4,16^ especially in root canals with larger buccolingual extensions.^2,4,7^ Higher percentage of voids in the root canal filling was observed for BioRoot RCS compared with GuttaFlow Bioseal (Coltene Whaledent, Langenau, Germany), in the cervical third of the root canals.^4^ Furthermore, a higher percentage of voids in the cervical/middle third was observed when using the single-cone technique in comparison with Tagger´s hybrid technique.^18^ Whereas a low percentage of voids has been reported for flattened root canals when filled with the single-cone technique using BCS or AHP.^2^ Up to now, there are still no studies in the Dental literature that evaluate the influence of sealer thickness on the behavior of endodontic materials under different pH conditions.

The aim of this study was to use micro-CT to evaluate the effect of immersion in an acidic (butyric acid – BA) or neutral (PBS) environment, on the physical behavior of BCS or AHPJ after filling oval root canals with different sealer thicknesses. The first null hypothesis was that the immersion solution would not influence the volumetric change, percentage of voids, and gaps at the material/dentin interface after filling oval root canals using BCS or AHPJ. The second null hypothesis was that the sealer thickness would not interfere with the properties of the materials, and the third null hypothesis was that there would be no difference between the variables relative to the time intervals of immersion in BA or PBS for both sealers.

## Methods

### Sample size calculation

The G*Power 3.1.7 program for Windows (Heinrich-Heine-Universitat Dusseldorf, Dusseldorf, Germany) was used for sample calculation. The One-Way ANOVA test was used with an alpha-type error of 0.05 and a beta power of 0.85 for all variables. Previous studies were used to determine the specific effect size for volumetric change, 0.98,^19^ percentage of voids, 0.71,^20^ and gaps at the interface, 1.76.^21^ A total of 8 specimens were indicated as the ideal size required.

### Sample selection

After approval by the Institutional Research Ethics Committee (Protocol CAAE No. 41926620.6.0000.5416), 32 extracted human teeth were selected, including maxillary second premolars, mandibular premolars, and distal roots of mandibular molars with single and oval root canals. Inclusion criteria were fully formed apices, no root fractures, and no signs of internal or external resorption. Teeth with an immature apex, curvatures, and previous endodontic treatment were excluded. To confirm these criteria, a digital radiography system (Kodak RVG 6100; Digital Radiography System, Marne-la-Vallée, France) and micro-CT with a voxel size of 35 µm (SkyScan 1272; Bruker, Kontich, Belgium) were used. The oval cross-section of the root canals was determined when the buccolingual diameter was equal to 2 or more times larger than the mesiodistal diameter^22^ at 9 mm from the radiographic apex,^2^ using the CTAn software (V1.15.4.0; SkyScan, Bruker, Kontich, Belgium). In addition, using the same software, the preoperative root canal volume (in mm^3^) was determined to ensure consistent volumes between the samples in the subsequent steps.

### Root canal preparation

After coronal access, the root canals were explored with 15 and 20 K-files (Dentsply, Maillefer, Ballaigues, Switzerland). The working length (WL) was determined to be 1 mm beyond the apical foramen. The roots were coated with condensation silicone (Oranwash, Zhermack SpA, Badia Polesine, Italy) to simulate the periodontal ligament and stabilize the samples during the procedures.^2,18^ Subsequently, a single operator , previously trained and calibrated, prepared all the root canals using the ProDesign Logic rotary system (Easy Equipamentos Odontológicas, Belo Horizonte, Brazil) 40/.05 at 950 rotations per minute (RPM) and 4 Newton centimeter (Ncm) driven by a VDW SILVER electric motor. (VDW GmbH, Munich, Germany). The instrument was introduced into the root canal using in-and-out movements up to the WL, and then 3 brushing movements against the buccal and lingual walls were performed. During the entire preparation, the root canals were irrigated with 5 mL of 1% sodium hypochlorite (NaOCl; Ciclo Farma, Serrana, Brazil) using a 5 mL syringe (Ultradent, South Jordan, UT) with a 30G Navitip needle (Ultradent). After root canal preparation, the 10 millimeters of the apical roots of each sample were sectioned using an Isomet 1000 cutter (Buehler Ltd., Lake Bluff, United States) to standardize the samples for further analysis. The specimens were finally irrigated with 5 mL of 2.5% NaOCl and 5 mL of 17% EDTA (Biodynamics, Ibiporã, Brazil) for 3 minutes, followed by 5 mL of distilled water.

The prepared root canals were identified and divided into two groups of 16 samples each, assigned to either BCS or AHPJ sealer, using simple stratified randomization considering the preoperative canal volume (mm^3^).

### Root canal filling

All root canals were filled using the single-cone technique with BCS or AHPJ sealer. Then 40/.04 gutta-percha master cones (Tanariman, Manacapuru, Brazil) were selected according to tip size and taper determine by using a Profilometer device (Profile Projector Nikon model 6C-2, Nikon, Tokyo, Japan). Digital radiographs were taken to confirm the adaptation of the cone at 1 mm beyond the apical foramen. For BCS (Lot: 64361), the sealer was injected into the root canals using syringes and plastic needles at 4 mm short of the WL according to the manufacturer’s instructions. The syringe plunger was lightly pressed until BCS flowed back into the cervical third, indicating complete filling of the root canals.^2,18,23^ For the root canals filled with AHPJ (Lot: 2205000392), 1g of the sealer was obtained after handling equal lengths of paste A and B mixed with a metal spatula (nº 24F; Duflex, Juiz de Fora, Brazil) on a slab , for 30 seconds until a consistent and homogeneous texture were obtained.^24^ The weight was confirmed by using a precision balance (HM-200; A&D Engineering, Inc., Bradford, USA). AHPJ was then applied to the canal using a size 40 Lentulo spiral (Dentsply Sirona) at low speed in a clockwise direction (Micromotor N270, Dabi-Atlante, Ribeirão Preto, Brazil), positioned 2 mm short of WL. A manual 40 K-file (Dentsply) was used with in-and-out movements to carry the sealer up to the WL. This procedure was repeated three times.^2^ The gutta-percha cone, evenly coated with a thin layer of sealer, was inserted into the canal, and the excess gutta-percha was cut and compacted vertically with a heat plugger (Golgran No. 2, São Caetano do Sul, Brazil). Digital radiographs in the buccolingual and mesiodistal directions were taken to evaluate the quality of root canal filling. The samples were stored in an oven at 37ºC and 95% humidity for 7 days to allow complete setting of the sealers.^2,18^

**Figure 1:**
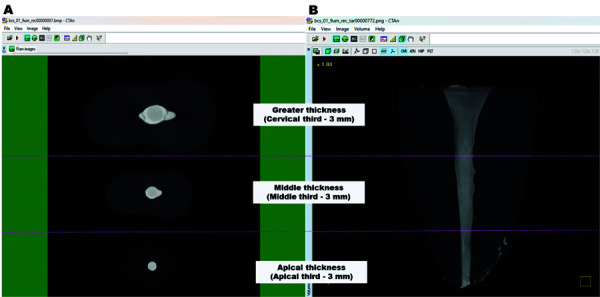
Representative axial and sagittal sections of micro-CT images of the sample division for analysis using the CTAn software. (A) Sealer thickness and (B) oval root canal thirds.

Filling of the root canals resulted in different sealer thicknesses throughout the root thirds [each 3 millimeters]: smaller thickness in the apical third, middle thickness in the middle third, and greater thickness in the cervical third (Figure 1A).

### Micro-CT scanning and immersion of the specimens

After filling, all samples were scanned with a micro-CT (SkyScan 1272; Bruker, Kontich, Belgium) using the following parameters: voxel size of 8.74 µm, rotation step 0.5º, frame averaging of 3, Aluminum filter 0.5 mm + Copper 0.038 mm, rotation 180°, 90 kilovolts (kV) and 110 microamperes (uA).

Before immersion, the specimens were divided into four groups using simple stratified randomization: BCS (BA or PBS) and AHPJ (BA or PBS) (n = 8). To ensure comparability between the experimental groups in terms of sealer thickness, the filling material volume (mm^3^) for each root third was calculated and analyzed using the ANOVA test (p > 0.05) (Table 1).

**Table 1:** Mean and standard deviation of the filling material volume (mm3) of the cervical, middle, and apical thirds.

Variables	Bio-C Sealer		AH Plus Jet	
	BA	PBS	BA	PBS
GT	3.23 ± 1.47	3.31 ± 1.41	3.88 ± 1.87	3.45 ± 1.10
Cervical third				
MDT	1.68 ± 0.96	1.62 ± 0.40	1.98 ± 0.81	1.86 ± 0.57
Middle third				
ST	0.98 ± 0.29	0.93 ±0.17	1.04 ± 0.14	1.12 ± 0.32
Apical third				

GT: greater thickness, MDT: middle thickness, ST: smaller thickness, BA: butyric acid, PBS: phosphate-buffered saline. There was no significant difference between the groups (p > 0.05).

Each specimen was individually and fully inserted into the Eppendorf tube with 1.5 mL of BA (Sigma Aldrich, Barueri, Brazil; pH: 4.1) or PBS (Sigma, pH: 7.0) and stored in an oven at 37ºC for 7 and 30 days. The BA solution was changed every 24 hours to ensure a stable acidic pH throughout the experiment.^13,25^ New micro-CT scans were performed after the different experimental time intervals using the same parameters previously described.

### Micro-CT analysis

Micro-CT image reconstructions at different experimental time intervals were performed using the NRecon software (V1.6.3; SkyScan, Bruker, Kontich, Belgium). 3D images were registered before and after immersion using the “3D registration” function in Data Viewer software (V1.5.1.; SkyScan, Belgium). Quantitative analyses were performed using CTAn software, to calculate the filling material volume (in mm^3^ and percentage). Additionally, the interface volume and void volume of the endodontic sealers were measured. Three-dimensional models and slices were created using the CTVox software (V.3.2; SkyScan, Belgium) and CTAn software.

Each third of the specimen was assessed since it was divided into 3-mm sections, based on the filling material and sealer thickness:

- smaller thickness (3 mm from the apical third);
- middle thickness (3 mm from the middle third);
- greater thickness (3 mm from the cervical third), as represented in Figure 1B.

After defining the top and bottom limits for each third, the volume of interest (VOI) was selected, excluding the dentin. Binarization of the samples was performed using a density histogram with adaptive thresholding. The total volume of the filling materials before and after immersion in BA or PBS over different time intervals was obtained by the 3D analysis in the CTAn software. The percentage of volumetric change was calculated using the following formula: [Percentage of volumetric change = (Filling material volume after immersion *100/Initial volume) -100].^5,25^ The percentage of voids was determined considering the percentage of filling material volume (sealer and gutta-percha) after the sealer set and after the experimental time intervals, using the formula: [Percentage of voids = 100 - percentage of filling material volume].^26^

The percentage of gaps at the material/dentin interface was calculated based on the methodology described by Gandolfi et al.^27^ The 3D distribution of gaps at the interface within the pre-defined VOI was calculated for each third. The limits (bottom and top) were determined, and adaptive thresholding was applied to recognize each object of interest during the binarization of the samples. After using the “Task List” with arithmetic and logical operations between the superimposed sections, a 3D-VOI was defined, representing the interface volume, which included part of the dentin and part of the filling material. Gaps with sizes starting at 8.74 μm within the 3D-VOI were detected.

**Figure 2:**
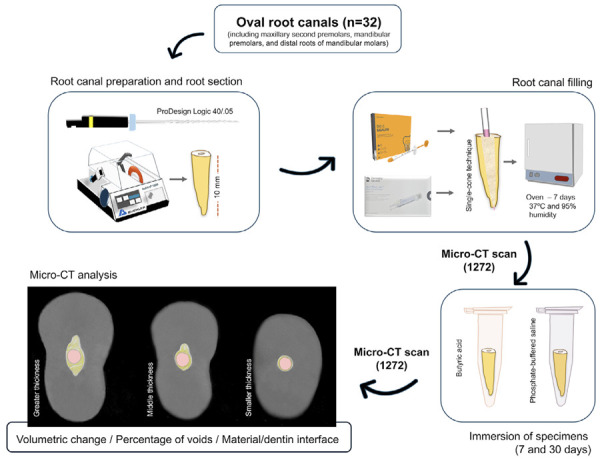
The experimental design workflow illustrates each phase conducted to assess the physical behavior of two endodontic materials with different sealer thicknesses after exposure to acidic or neutral environments.

Figure 2 illustrates the experimental design workflow for evaluating the physical behavior of two endodontic materials with different sealer thicknesses after exposure to acid or neutral environment.

### Statistical analysis

All data were subjected to the Shapiro-Wilk test. Data relative to the percentage of volumetric change and percentage of voids presented a non-normal distribution. Kruskal-Wallis and Dunn tests were used to compare groups. The Friedman and Wilcoxon Paired tests were used to compare immersion time intervals. Data relative to the filling material volume of each third, and the percentage of gaps at the material/dentin interface showed a normal distribution. One-way ANOVA and Tukey tests were used for comparisons between groups. Repeated measures ANOVA and paired Tukey tests were used for comparisons among immersion periods. The level of significance was 5% for all analyses.

## Results

The filling material volume (mm^3^) in each root third was comparable throughout all groups, confirming the homogeneity of the samples (p > 0.05) (Table 1). This finding demonstrates that the sealer thicknesses were standardized, ensuring the absence of significant discrepancies that could affect the reliability of the results.

In the time intervals of 7 days, BCS and AHPJ showed similar volumetric changes and percentages of gaps at the interface irrespective of the immersion solution and sealer thickness (p > 0.05). After 30 days, BA increased the volumetric loss and presence of voids and gaps at the material/dentin interface of both materials (p < 0.05) (Tables 2, 3, and 4).

**Table 2:** Median, minimum, and maximum values of the percentage of volumetric change of Bio-C Sealer or AH Plus Jet after immersion in butyric acid (BA) or PBS for 7 and 30 days

Variables	Bio-C Sealer		AH Plus Jet	
	BA	PBS	BA	PBS
Immersion time interval 7 days				
GT	-1.23 (-3.36-(-0.58))^aA*^	-0.36 (-2.40-1.40)^aA^	-0.88 (-4.54-0.28)^aA*^	1.27 (-2.90-2.67)^aA^
Cervical third				
MDT	-0.21 (-3.31-0.24)^aA^	-0.04 (-1.71-1.62)^aA^	-1.10 (-1.07-(-0.04))^aA^	-0.99 (-2.63-1.31)^aA^
Middle third				
ST	-2.00 (-9.93-(-0.26))^aA^	-0.28 (-3.97-0.94)^aA^	-2.44 (-4.20-0.45)^aA*^	-1.17 (-3.09-1.14)^aA^
Apical third				
Immersion time interval 30 days				
GT	-4.58 (-5.92-(-2.87))^bB*^	-0.77 (-3.05-1.11)^aA^	-3.77 (-10.46-(-2.28))^bB*^	-1.59 (-4.89-1.91)^aA^
Cervical third				
MDT	-1.22 (-3.80 – 0.89)^abA^	-0.83 (-2.44-1.20)^bA^	-1.72 (-4.27-(-0.97))^aA^	0.15 (-3.54-5.69)^abA^
Middle third				
ST	-3.30 (-11.78-(-0.35))^bB^	1.78 (-5.76-2.96)^aA^	-4.00 (-5.39-(-1.64))^bB*^	-0.55 (-4.70-3.10)^abA^
Apical third				

GT: Greater thickness, MDT: middle thickness, ST: smaller thickness, PBS: phosphate-buffered saline. Negative values: volumetric loss, Positive values: volumetric gain. Different superscript lowercase letters in the same line indicate a significant difference between groups (p < 0.05). Different superscript capital letters in the same column indicates a significant difference between the thirds of each group (p < 0.05). *indicates a significant difference between immersion periods (p < 0.05).

**Table 3:** Median, minimum, and maximum values of the percentage of voids of Bio-C Sealer or AH Plus Jet after immersion in butyric acid (BA) or PBS for 7 and 30 days.

Variables	Bio-C Sealer		AH Plus Jet	
	BA	PBS	BA	PBS
Initial period				
GT	0.64 (0.09-1.92)^bAB*^	0.33 (0.13-5.78)^bA*^	2.30 (0.20-14.77)^aA*^	1.88 (0.05-6.95)^abA^
Cervical third				
MDT	0.30 (0.01-0.82)^aB^	0.47 (0.01-0.78)^aA^	0.77 (0.01-6.02)^aAB*^	0.60 (0.01-4.64)^aAB^
Middle third				
ST	0.89 (0.07-1.98)^aA*^	0.25 (0.06-0.78)^bA^	0.26 (0.06-0.79)^bC*#^	0.35 (0.01-2.70)^bB*^
Apical third				
Immersion period 7 days				
GT	1.26 (0.69-2.20)^aA*^	0.59 (0.13-5.80)^aA^	2.64 (0.23-6.77)^aA#^	1.82 (0.23-7.16)^aA^
Cervical third				
MDT	0.28 (0.06-0.99)^aB^	0.56 (0.17-1.81)^aA^	0.98 (0.02-5.23)^aAB#^	0.16 (0.02-4.79)^aA^
Middle third				
ST	1.20 (0.55-1.85)^aC#^	0.39 (0.09-3.60)^aA^	0.59 (0.12-1.32)^aB*^	0.58 (0.02-2.91)^aA*^
Apical third				
Immersion period 30 days				
GT	3.37 (2.02-9.38)^aA*^	0.92 (0.22-6.65)^bA*^	5.53 (0.36-8.18)^aA*#^	2.31 (0.01-9.21)^abA^
Cervical third				
MDT	0.39 (0.10-2.17)^bB^	0.71 (0.03-1.93)^bA^	2.26 (0.10-6.02)^aAB*#^	0.58 (0.01-4.82)^bAB^
Middle third				
ST	1.98 (1.24-5.16)^aA*#^	0.24 (0.01-2.96)^bA^	1.24 (0.11-1.99)^abB#^	0.41 (0.07-3.61)^cB^
Apical third				

GT: Greater thickness, MDT: middle thickness, ST: smaller thickness, PBS: phosphate-buffered saline. Different superscript lowercase letters in the same line indicate a significant difference between groups (p < 0.05). Different superscript capital letters in the same column indicate a significant difference between the thirds of each group (p < 0.05). ^*#^Similar symbols indicate significant differences between immersion periods (p < 0.05).

**Table 4:** Mean and standard deviation of the percentage of gaps at the material/dentin interface of Bio-C Sealer and AH Plus Jet after immersion in butyric acid (BA) or PBS for 7 and 30 days

Variables	Bio-C Sealer		AH Plus Jet	
	BA	PBS	BA	PBS
Initial period				
GT	5.77 ± 2.19^aA*^	5.67 ± 2.23^aA^	7.69 ± 2.53^aA*^	8.55 ± 3.16^aA^
Cervical third				
MDT	4.68 ± 2.09^aA*^	4.76 ± 2.14^aA^	7.07 ± 2.16^aA*^	6.86 ± 2.39^aA^
Middle third				
ST	5.73 ± 2.10^aA*^	4.63 ± 2.03^aA*^	6.54 ± 1.54^aA*^	6.13 ± 1.60^aA*^
Apical third				
Immersion time interval 7 days				
GT	7.16 ± 2.25^aA*^	6.33 ± 2.30^aA^	9.27 ± 3.94^aA*^	9.79 ± 3.43^aA^
Cervical third				
MDT	5.93 ± 2.06^aA*^	5.47 ± 2.28^aA^	8.34 ± 2.48^aA^	8.30 ± 2.38^aA^
Middle third				
ST	6.93 ± 2.34^aA#^	5.62 ± 1.87^aA^	8.12 ± 1.75^aA*^	7.27 ± 1.98^aA^
Apical third				
Immersion time interval 30 days				
GT	10.03 ± 2.85^abA*^	6.42 ± 1.97^bA^	11.89 ± 4.04^aA*^	10.29 ± 4.73^abA^
Cervical third				
MDT	6.92 ± 1.76^aB*^	5.59 ± 2.18^aA^	9.18 ± 2.87^aA*^	7.84 ± 3.61^aA^
Middle third				
ST	8.90 ± 2.13^aAB*#^	5.82 ± 1.88^bA*^	9.70 ± 1.85^aA*^	7.94 ± 1.96^abA*^
Apical third				

GT: Greater thickness, MDT: middle thickness, ST: smaller thickness, PBS: phosphate-buffered saline. Different superscript lowercase letters in the same line indicate a significant difference between groups (p < 0.05). Different superscript Capital letters in the same column indicate a significant difference between the thirds of each group (p < 0.05). ^*#^Similar symbols indicate significant differences between immersion periods (p < 0.05).

**Figure 3:**
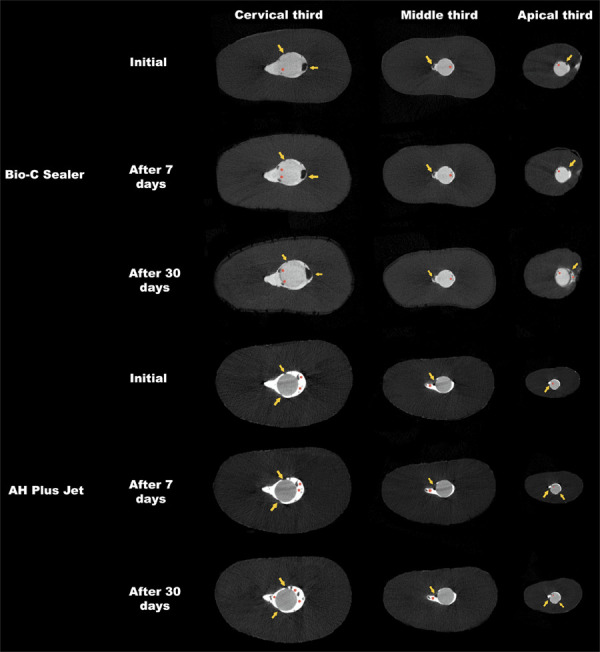
Cross-sections (axial) of the two oval root canals scanned with micro-CT showing the voids (red asterisks) and gaps at the material/dentin interface (yellow arrows) of Bio-C Sealer or AH Plus Jet before and after immersion in butyric acid for 7 and 30 days.

**Figure 4:**
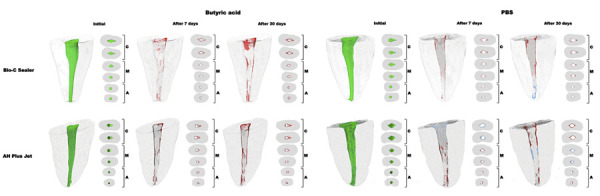
Three-dimensional images and cross-sectional views of the cervical (C), middle (M), and apical (A) thirds of 4 oval root canals showing the initial volume (green), volume gain (blue), and volume loss (red) of the Bio-C Sealer or AH Plus Jet before and after immersion in butyric acid or phosphate-buffered saline (PBS) for 7 and 30 days.

Relative to sealer thickness, a higher presence of voids was observed in the greater thickness compared with the smaller thickness in both sealers after 7 days (p < 0.05) (Figure 3). Immersion in BA for 30 days resulted in greater volume loss in the greater and smaller thicknesses for both materials compared with the middle thickness (p < 0.05) (Figure 4). Furthermore, BCS showed a higher percentage of gaps at the material/dentin interface in the greater thickness compared with the middle thickness (p < 0.05). The greater sealer thickness for AHPJ showed a higher percentage of voids compared with the smaller thickness without the influence of the immersion solution after 30 days (p < 0.05) (Figure 3).

Concerning the immersion time intervals, higher volumetric loss was reported for AHPJ in the greater and smaller thickness and for BCS in the greater thickness after 30 rather than 7 days in BA (p < 0.05) (Figure 4 and Table 2). The baseline showed a lower percentage of voids and gaps at the interface when compared with the immersion time intervals of 7 and 30 days (p < 0.05) (Figure 3).

## Discussion

A three-dimensional filling is essential for the success of endodontic treatment.^2,18^ However, the acidic pH resulting from persistent periapical infections or exposure to oral cavity fluids can compromise the chemical, physical, and mechanical properties of endodontic sealers.^11-15^. Moreover, the quality of filling is influenced by the morphology of root canals.^2,6^ Therefore,, in this study, root canals with an oval cross-section were used as they represent a real challenge to achieving adequate filling, in clinical practice.^1,6,7^ In order to simulate a scenario close to clinical situations, BA was used, as it represents one of the metabolic by-products of anaerobic bacteria present in inflammation.^28^ PBS was considered a comparison parameter as it mimics a neutral environment.^1,5,9^ Furthermore, the variables could be quantified by mean of analyses using high-resolution micro-CT images in relation to volumetric behavior and the presence of voids and gaps at the material/dentin interface of endodontic sealers after the different experimental time intervals of the present study.^1-8,11,17-20^ Our null hypotheses were rejected since the time interval of immersion, the pH of the solution as well as the sealer thickness had effect on the properties valuated .

Significant volumetric loss and a higher percentage of voids and gaps at the material/dentin interface were observed for both materials after exposure to acidic pH for 30 days. These results may be related to inhibition of the hydration and setting reactions of these materials, and less deposition of carbonated apatite on the surface and interface of the material/dentin. The hydrophilic particles of calcium silicate-based sealers have greater contact with liquid molecules.^29^ Thus, the increase in water sorption (absorption and adsorption of liquids) and solubility (loss of mass) can affect the dimensional stability of materials^30^ and reduce the filling capacity.^31^ Furthermore, structural changes in hydroxyapatite crystals after acid challenge cause greater porosity and solubility of bioceramic materials.^1^ The corrosive effect of the acidic environment and the exposure time may also explain the results observed for the degradation of the AHPJ sealer.^32^

Longer periods of assessment of the physical properties of endodontic materials are essential, considering that their hydration reaction continues even after the final setting time.^29^ A previous study showed higher volumetric loss and a lower percentage of voids for BCS when immersed in distilled water for 7 days.^5^ In the present study, higher volumetric loss and a higher percentage of voids and gaps at the material/dentin interface of BCS and AHPJ were observed over a time interval of 30 days in comparison with 7 days. This finding may be related to the acidic environment and the prolonged period of exposure. In agreement with our results, a previous study reported greater volumetric loss for EndoSequence BC Sealer after 30 days in acidic pH.^1^

When evaluating the sealer thickness on the properties of BCS or AHPJ, the greater thickness of both sealers negatively affected the properties evaluated irrespective of the time interval. Root canals have a flattened cross-section in the cervical third.^2^ Thus, greater sealer thickness may favor the presence of voids and gaps at the material/dentin interface. In agreement with our findings, lower filling capacity in the cervical third was observed when calcium silicate-based sealers were used.^4^ However, contrary to our results, a previous study showed a lower percentage of voids for BCS and AHPJ, in addition to a low percentage of gaps at the interface for BCS after filling oval root canals using the continuous condensation wave technique or the Tagger’s hybrid technique.^7^ This difference was possibly related to the filling technique used in the present study. The single-cone technique requires a larger amount of sealer,^2,3^ which may have negatively affect the volumetric behavior of the material. Furthermore, the hydrophobic nature of gutta-percha may negatively affect its interaction with the material, considering the hydrophilic nature of calcium silicate-based sealers.^6^ In addition, the greater thickness of the AHPJ sealer presented a higher percentage of voids compared with the smaller thickness, irrespective of the immersion solutions. Although AHPJ presents a dedicated Auto Mix Tip, which automatically mixes the sealer components, this method can interfere with its homogenization and consequently affect its physicochemical and mechanical properties.^24^ Therefore, AHPJ was manually mixed in the present study.

In this study, the cervical and apical thirds were kept in direct contact with the immersion environment, differently from middle third, thus explaining its superior performance compared with cervical and apical thirds. The apical third exhibited fewer changes in its properties than the cervical third. This result may be related to the reduced sealer thickness and the improved adaptation of the gutta-percha point in this region. Root canal filling strategies prioritize maximizing gutta-percha mass while minimizing sealer volume,^4^ particularly in root canals with greater buccolingual extensions.^7,18^ Previous studies have indicated that thermoplastic techniques enhance filling quality in oval and flattened root canals.^7,16,18^Therefore, our findings underscore the critical role of sealer thickness in filling quality, suggesting that a smaller thickness is preferable, especially in acidic environments.

Despite limitations of the experimental ex vivo design, our findings indicated that sealer selection, sealer thickness, and pH play an essential role in the integrity and stability of root canal filling. Further studies should incorporate different root anatomical configurations, filling techniques, immersion environments and in vivo models. These approaches will enhance the understanding of how these variables influence long-term treatment outcomes in different clinical scenarios.

## Conclusions

Within the limitations of this ex-vivo study, it can be concluded that an acidic environment impairs the properties of Bio-C Sealer and AH Plus Jet, especially after immersion for 30 days. The greater sealer thickness in the cervical third negatively affects the quality of the filling for both sealers.

## Data Availability

The datasets generated during and/or analyzed during the current study are available from the corresponding author on reasonable request.
